# Role of NAFLD on the Health Related QoL Response to Lifestyle in Patients With Metabolic Syndrome: The PREDIMED Plus Cohort

**DOI:** 10.3389/fendo.2022.868795

**Published:** 2022-06-29

**Authors:** Diego Martínez-Urbistondo, Rodrigo San-Cristóbal, Paula Villares, Miguel Ángel Martínez-González, Nancy Babio, Dolores Corella, José Luis del Val, José Ma Ordovás, Ángel M. Alonso-Gómez, Julia Wärnberg, Jesús Vioque, Dora Romaguera, José López-Miranda, Ramon Estruch, Francisco J. Tinahones, José Lapetra, J. Luís Serra-Majem, Aurora Bueno-Cavanillas, Josep A. Tur, Alba Marcos, Xavier Pintó, Miguel Delgado-Rodríguez, Pilar Matía-Martín, Josep Vidal, Clotilde Vázquez, Emilio Ros, María Vanessa Bullón Vela, Antoni Palau, Jose V. Sorli, Marta Masagué, Itziar Abete, Anai Moreno-Rodríguez, Inma Candela-García, Jadwiga Konieczna, Antonio García-Ríos, Oscar Lecea Juárez, Olga Portolés, Paco Martín, Albert Goday, M Ángeles Zulet, Jessica Vaquero-Luna, María del Carmen Sayón Orea, Isabel Megías, Enric Baltasar, J. Alfredo Martínez, Lidia Daimiel

**Affiliations:** ^1^ Internal Medicine Department, Hospital HM Sanchinarro, HM Hospitales, Madrid, Spain; ^2^ Cardiometabolic Nutrition Group, Precision Nutrition and Cardiometabolic Health Program, Instituto Madrileño de Estudios Avanzados (IMDEA) Food, Centro de Excelencia en Investigación (CEI) Universidad Autónoma de Madrid (UAM) + Centro Superior de Investigaciones Científicas (CSIC), Madrid, Spain; ^3^ Department of Preventive Medicine and Public Health, University of Navarra, IdiSNA, Pamplona, Spain; ^4^ Consorcio CIBER, M.P. Fisiopatología de la Obesidad y Nutrición (CIBERObn), Instituto de Salud Carlos III (ISCIII), Madrid, Spain; ^5^ Department of Nutrition, Harvard T. H. Chan School of Public Health, Boston, MA, United States; ^6^ Universitat Rovira i Virgili, Departament de Bioquímica i biotecnologia, Unitat de Nutrició Humana, Reus, Spain; ^7^ Institut d’Investigació Sanitària Pere Virgili (IISPV), Hospital Universitari San Joan de Reus. Human Nutrition unit, Reus, Spain; ^8^ Department of Preventive Medicine, University of Valencia, Valencia, Spain; ^9^ Cardiovascular Risk and Nutrition Research Group (CARIN), Hospital del Mar Medical Research Institute (IMIM), Barcelona, Spain; ^10^ Nutritional Genomics and Epigenomics Group, Precision Nutrition and Obesity Program. Instituto Madrileño de Estudios Avanzados (IMDEA) Food, Centro de Excelencia en Investigación (CEI) Universidad Autónoma de Madrid (UAM) + Centro Superior de Investigaciones Científicas (CSIC), Madrid, Spain; ^11^ Nutrition and Genomics Laboratory, Jean Mayer United States Department of Agriculture (JM_USDA) Human Nutrition Research Center on Aging, Tufts University, Boston, MA, United States; ^12^ Bioaraba Health Research Institute, Osakidetza Basque Health Service, Araba University Hospital, University of the Basque Country Universidad del País Vasco/Euskal Herriko Unibertsitatea (UPV/EHU), Vitoria-Gasteiz, Spain; ^13^ Department of Nursing, School of Health Sciences, University of Málaga, Instituto de Investigación Biomédica de Málaga (IBIMA), Málaga, Spain; ^14^ Centro de Investigación Biomédica en Red de Epidemiología y Salud Pública (CIBERESP), Instituto de Salud Carlos III, Madrid, Spain; ^15^ Instituto de Investigación Sanitaria y Biomédica de Alicante. Universidad Miguel Hernández (ISABIAL-UMH), Alicante, Spain; ^16^ Research Group on Nutritional Epidemiology & Cardiovascular Physiopathology (NUTRECOR). Health Research Institute of the Balearic Islands (IdISBa), University Hospital Son Espases (HUSE), Palma de Mallorca, Spain; ^17^ Lipids and Atherosclerosis Unit, Department of Internal Medicine, Maimonides Biomedical Research Institute of Cordoba (IMIBIC), Reina Sofia University Hospital, University of Cordoba, Córdoba, Spain; ^18^ Department of Internal Medicine, Instituto de Investigaciones Biomédicas August Pi i Sunyer (IDIBAPS), Hospital Clinic, University of Barcelona, Barcelona, Spain; ^19^ Department of Endocrinology, Instituto de Investigación Biomédica de Málaga (IBIMA), Virgen de la Victoria Hospital, University of Málaga, Málaga, Spain; ^20^ Department of Family Medicine, Research Unit, Distrito Sanitario Atención Primaria Sevilla, Sevilla, Spain; ^21^ Research Institute of Biomedical and Health Sciences Instituto Universitario de Investigaciones Biomédicas y Sanitarias (IUIBS), University of Las Palmas de Gran Canaria, Preventive Medicine Service, Centro Hospitalario Universitario Insular Materno Infantil (CHUIMI), Canarian Health Service, Las Palmas, Spain; ^22^ Department of Preventive Medicine and Public Health, University of Granada, Granada, Spain; ^23^ Research Group on Community Nutrition & Oxidative Stress, University of Balearic Islands, Palma de Mallorca, Spain; ^24^ Institute of Biomedicine (IBIOMED), University of León, León, Spain; ^25^ Lipids and Vascular Risk Unit, Internal Medicine, Hospital Universitario de Bellvitge, Hospitalet de Llobregat, Barcelona, Spain; ^26^ Departamento de Ciencias de la Salud, Centro de Estudios Avanzados en Olivar y Aceites de Oliva, Universidad de Jaén, Jaén, Spain; ^27^ Department of Endocrinology and Nutrition, Instituto de Investigación Sanitaria Hospital Clínico San Carlos (IdISSC), Madrid, Spain; ^28^ Biomedical Research Centre for Diabetes and Metabolic Diseases Network (CIBERDEM), Instituto de Salud Carlos III (ISCIII), Madrid, Spain; ^29^ Endocrinology and Nutrition Service, Instituto de Investigaciones Biomédicas August Pi i Sunyer (IDIBAPS), Hospital Clinic, University of Barcelona, Barcelona, Spain; ^30^ Department of Endocrinology and Nutrition, Hospital Fundación Jimenez Díaz, Instituto de Investigaciones Biomédicas IISFJD. University Autónoma, Madrid, Spain; ^31^ Department of Nutrition, Food Sciences and Physiology, University of Navarra, Pamplona, Spain; ^32^ Centro de Salud San Pola, Alicante, Spain; ^33^ Departament de Medicina, Universitat Autònoma de Barcelona, Barcelona, Spain; ^34^ Nutritional Control of the Epigenome Group. Precision Nutrition and Obesity Program. Instituto Madrileño de Estudios Avanzados (IMDEA) Food, Centro de Excelencia en Investigación (CEI) Universidad Autónoma de Madrid (UAM) + Centro Superior de Investigaciones Científicas (CSIC), Madrid, Spain

**Keywords:** NAFLD, metabolic syndrome, mediterranean diet, physical activity, HRQoL

## Abstract

**Objective:**

To evaluate the effect of Non-alcoholic fatty liver disease (NAFLD) status in the impact of lifestyle over Health-related quality of life (HRQoL) in patients with metabolic syndrome (MetS).

**Methods:**

Baseline and 1 year follow up data from the PREDIMED-plus cohort (men and women, 55-75 years old with overweight/obesity and MetS) were studied. Adherence to an energy-restricted Mediterranean Diet (er-MeDiet) and Physical Activity (PA) were assessed with a validated screeners. Hepatic steatosis index (HSI) was implemented to evaluate NAFLD while the SF-36 questionnaire provided HRQoL evaluation. Statistical analyses were performed to evaluate the influence of baseline NAFLD on HRQoL as affected by lifestyle during 1 year of follow up.

**Results:**

Data from 5205 patients with mean age of 65 years and a 48% of female participants. Adjusted linear multivariate mixed regression models showed that patients with lower probability of NAFLD (HSI < 36 points) were more responsive to er-MeDiet (β 0.64 vs β 0.05 per er-MeDiet adherence point, p< 0.01) and PA (β 0.05 vs β 0.01 per MET-h/week, p = 0.001) than those with high probability for NAFLD in terms Physical SF-36 summary in the 1 year follow up. 10 points of er-MeDiet adherence and 50 MET-h/week were thresholds for a beneficial effect of lifestyle on HRQoL physical domain in patients with lower probability of NAFLD.

**Conclusion:**

The evaluation of NAFLD by the HSI index in patients with MetS might identify subjects with different prospective sensitivity to lifestyle changes in terms of physical HRQoL (http://www.isrctn.com/ISRCTN89898870).

## Introduction

The prevalence of Non-alcoholic Fatty Liver Disease (NAFLD) is raising worldwide (1). Indeed, lipid metabolism associated disorders are the most rapidly increasing cause of morbidity and mortality among hepatic patients in Western countries (2). NAFLD can be considered a manifestation of the metabolic syndrome (MetS) burden in the liver (3, 4). In terms of pathogenesis, fatty liver disease is the result of the clustering of genetic inheritance, lifestyle factors, ageing, hepatotoxic drugs, co-morbidities and gut microbiota (5–11).

In this context, non-invasive assessment of liver steatosis using indexes, such as Hepatic Steatosis Index (HSI) were developed to select patients at a higher risk of NAFLD in population studies (12). These scales could provide a non-invasive longitudinal monitoring of patients and an objective quantitation of the effect of therapeutic measures on NAFLD status.

Weight loss is a major target in patients with fatty liver disease (13). In this context, changes in lifestyle have an impact on NAFLD status (7). Among dietary patterns, the Mediterranean Diet (MedDiet) provides a balanced nutrient composition, which should be strongly deemed in treating this disease (14). Furthermore, moderate physical activity (PA) has shown a healthy potential in the reduction of NAFLD morbid severity through the improvement of insulin resistance, anti-inflammatory effects and antioxidant mechanisms.

Likewise, both MedDiet and PA are closely related to quality of life (QoL) in patients with MetS (15, 16). The SF-36 index is widely used, being sensitive to changes and has been validated for the Spanish population, separating information through mental and physical components of QoL and providing a precision medicine approach to Health-Related Quality of life (HRQoL). The physical aggregated component of SF-36 (PCS) mainly accounts for categories related to physical function, physical role, vitality, body pain and general health perceptions. Thus, the PCS is relevant in the clinical setting for the screening of physical factors that influence medical issues in QoL (17, 18).

The personalization of indications and the linkage between lifestyle intervention and a short-term benefit in terms of quality of life could improve the adherence to healthy habits, which may benefit NAFLD patients. In this context, the objective of the present study is the longitudinal evaluation of the impact of MedDiet and Physical activity on Health-related Quality of Life according to a baseline non-invasive evaluation of the NAFLD status based on the HSI score in patients with MetS from the PREDIMED-Plus trial.

## Methods

### Study Design and Participants

This analysis was based on baseline and 1 year data of the multicenter PREDIMED-Plus trial. The study protocol, including study design and data collection, has been published (19) and can be found at the PREDIMED-Plus website (https://www.predimedplus.com/en/). The detailed description of design and aims of the study could be found in **Appendix 1**. All authors had access to the study data and had reviewed and approved the final manuscript.

### Hepatic Steatosis Index

The liver status in terms of risk of NAFLD was measured with the Hepatic Steatosis Index (HSI), which was calculated using the described formula:


   (1) $$HSI\;=\;8\;x\frac{ALT}{AST}+\;BMI\left(+\;2\;if\;type\;2\;diabetes\;yes,\;+\;2\;if\;female\right)$$

A cut-off value of 36 points was used to select patients with a higher risk of NAFLD as described in previous reports (20).

### Health-Related Quality of Life

An adapted version of previously published 36-items HRQL questionnaire (SF36-HRQL) was used. This scale is validated for the Spanish population and extensively used to measure the subjective awareness of health and capability or physical constraints to manage with daily tasks. The PCS SF-36 index was calculated according to previously published coefficients (18).

### Statistical Analyses

**Figure 1:**
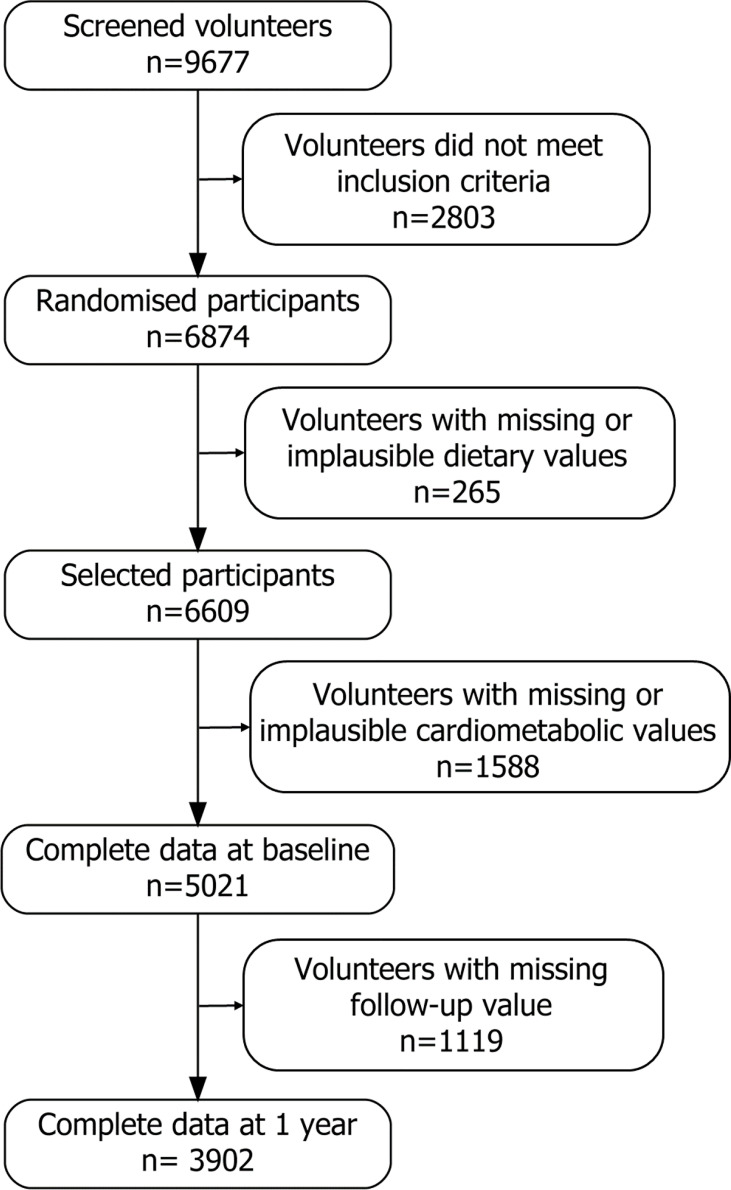
Flowchart of the PREDIMED-Plus participants.

Data from 5021 participants were included (**Figure 1**). The patient’s characteristics were defined as mean ± SD for quantitative variables and as proportions for qualitative variables. Participants were stratified according to the HSI quartiles and regarding the 36-point cut-off at baseline. The score for each category of the SF36-HRQL tests as well as for the physical component dimension were evaluated as continuous variables. Data among quartiles were compared using ANOVA. For longitudinal analyses, data from volunteers with missing or implausible values for the defined variables at 1 year of follow-up were excluded, including 3902 participants (**Figure 1**). Longitudinal analysis for the variation of the SF-36 components were analyzed with correlation panels. Afterwards, linear mixed model with individual random effect adjusted by age, sex, recruitment center, daily alcohol consumption, total caloric intake, waist circumference and number of metabolic syndrome features were applied to evaluate and compare the response of SF-36 PCS depending on er-MeDiet and PA adherence in both HSI < 36 and HSI ≥ 36 subgroups. Contrasts were performed including the interaction between modifiable factors (Mediterranean diet adherence and physical activity) as an independent variable in the model. Two-tailed level of 0.05 were considered as a threshold for statistical significance. All analyses were conducted with data from database version 202012220958_PREDIMEDplus_2anys_2020-12-22. “R-Studio1.4.17” (RStudio Team. PBC, Boston, MA) was used for analysis.

## Results

### Population Description and Liver Status Assessment at Baseline

A total of 5,021 patients fulfilled the inclusion criteria and were analyzed in the study (**Figure 1**). At baseline, the mean age of the study sample was 65.10 ± 4.91 years with a 48.1% of female participants. Demographic, anthropometric, lifestyle characteristics and MetS features are described by HSI quartiles (**Table 1**) and by HSI validated cut-off values HSI >36 and HSI < 36 (**Supplementary Table 1**). As expected, participants with higher HSI index showed higher levels of glucose, tryglicerides and liver enzymes. They also showed higher BMI and waist perimeter and lower HDL levels. They also showed a worse lifestyle indicated by a lower adherence to a MedDiet, lower PA and higher sedentarism. A poor control of the risk factors included in MetS was found to be directly related to the HSI score with statistical signification, except for hypertension (p < 0.05) (**Supplementary Figure 1**).

**Table 1:** Population characteristics at baseline.

Variable	Global	HSI p < 25	HSI p26 - p50	HSI p51 – p75	HSI > p75	p
**HSI interval [min - max]**		[30.95 - 39.62]	[39.62 - 42.68]	[42.69 - 46.31]	[46.31 - 192.54]	NA
**n**	5021	1256	1255	1255	1255	
**Age, years (SD)**	65.10 (4.91)	65.48 (5.08)	65.63 (4.88)	64.76 (4.91)	64.53 (4.71)	**<0.001**
**Sex, female (%)**	2417 (48.1)	455 (36.2)	565 (45.0)	657 (52.4)	740 (59.0)	**<0.001**
**Civil status (%)**						0.052
** Single**	248 (4.9)	61 (4.9)	56 (4.5)	61 (4.9)	70 (5.6)	
** Married**	3857 (77.0)	989 (78.9)	970 (77.5)	977 (78.0)	921 (73.6)	
** Widowed/divorced**	906 (18.1)	204 (16.3)	226 (18.1)	215 (17.2)	261 (20.8)	
**Education level (%)**						**0.001**
** Primary**	2479 (49.4)	570 (45.4)	606 (48.3)	657 (52.4)	646 (51.5)	
** Secondary**	1461 (29.1)	369 (29.4)	373 (29.7)	352 (28.0)	367 (29.2)	
** College**	1081 (21.5)	317 (25.2)	276 (22.0)	246 (19.6)	242 (19.3)	
**Working status, inactive (%)**	4005 (80.1)	1005 (80.3)	1017 (81.2)	974 (78.0)	1009 (80.7)	0.192
**Body mass index, kg/m^2^, (SD)**	32.51 (3.42)	29.20 (1.58)	31.23 (1.98)	33.26 (2.27)	36.36 (2.71)	**<0.001**
**Waist circumference, cm, (SD)**	107.48 (9.55)	101.21 (7.36)	105.08 (7.85)	108.91 (8.45)	114.71 (8.83)	**<0.001**
**Adherence to MeDiet, 0-17, (SD)**	8.48 (2.64)	8.57 (2.73)	8.60 (2.64)	8.45 (2.62)	8.33 (2.58)	**0,042**
**Physical activity, METs-min/week, (SD)**	2519.35 (2322.82)	2972.26 (2457.73)	2625.55 (2299.09)	2470.96 (2392.26)	2008.27 (2016.13)	**<0.001**
**Sedentarism, Yes (%)**	2193 (43.7)	462 (36.8)	495 (39.5)	581 (46.3)	655 (52.2)	**<0.001**
**Energy intake reported, Kcal/day, (SD)**	2351.48 (552.16)	2383.52 (550.77)	2354.77 (541.46)	2322.94 (557.01)	2344.64 (558.17)	0.05
**Alcohol intake, g/day, (SD)**	10.90 (14.86)	12.23 (15.08)	11.19 (14.89)	10.92 (15.25)	9.27 (14.08)	**<0.001**
**Fasting blood glucosa, mg/dl, (SD)**	113.44 (27.30)	105.11 (19.19)	110.90 (25.18)	115.80 (28.37)	121.97 (31.98)	**<0.001**
**Fasting blood tryglicerides, mg/dl, (SD)**	140.88 (52.93)	132.94 (52.38)	138.51 (51.98)	145.23 (53.09)	146.83 (53.15)	<0.001
**Fasting High-density lipoprotein, mg/dl, (SD)**	48.01 (11.63)	48.86 (12.49)	47.86 (10.95)	47.67 (11.12)	47.65 (11.88)	**0,027**
**Alanin aminotransferase, UI/L, (SD)**	26.71 (15.05)	20.36 (8.19)	24.39 (12.83)	28.24 (14.35)	33.86 (19.24)	**<0.001**
**Aspartate aminotransferase, UI/L, (SD)**	23.10 (9.52)	22.72 (8.54)	22.68 (9.05)	23.57 (10.06)	23.42 (10.30)	**0,029**
**Hepatic steatosis index, points (SD)**	43.28 (5.80)	37.41 (1.63)	41.15 (0.88)	44.42 (1.05)	50.15 (6.53)	**<0.001**
**Systolic blood pressure (mmHg)**	139.72 (16.87)	138.51 (16.59)	140.00 (17.23)	139.65 (16.70)	140.74 (16.91)	**0.01**
**Diastolic blood pressure (mmHg)**	80.64 (9.86)	80.46 (9.05)	80.54 (9.99)	80.94 (10.06)	80.62 (10.30)	0.644
**Diagnosed hypertension (%)**	4187 (84.0)	1037 (83.3)	1037 (83.0)	1036 (83.5)	1077 (86.3)	0.085
**Diagnosed diabetes (%)**	1358 (27.1)	176 (14.1)	289 (23.1)	388 (31.0)	505 (40.3)	**<0.001**
**Liver esteatosis HSI > 36 points (%)**	4777 (95.1)	1012 (80.6)	1255 (100.0)	1255 (100.0)	1255 (100.0)	**<0.001**

HSI, Hepatic steatosis index; SD, Standard deviation; MeDiet, Mediterranean Diet.

Bold for p values < 0.05.

NA, Not applicable.

### Health Related Quality of Life Distribution at Baseline

The levels of HRQoL were described as the result of the 8 categories of SF-36 and the 2 validated physical and mental summaries among HSI quartiles. These results are shown divided by HSI quartiles (**Table 2**). Quality of life showed a statistically significant inverse association to HSI score quartile among all categories and both summaries (p< 0,001).

**Table 2:** Score of the SF-36 Quality of Life categories among HSI baseline levels.

Variable (0-100 points)	HSI p < 25	HSI p26 - p50	HSI p51 – p75	HSI > p75	p
**HSI interval [min - max]**	[30.95 - 39.62]	[39.62 - 42.68]	[42.69 - 46.31]	[46.31 - 192.54]	NA
**Physical status, (SD)**	82.01 (16.78)	77.43 (18.84)	75.57 (18.75)	69.12 (21.64)	**<0.001**
**Physical role, points, (SD)**	81.28 (32.79)	77.23 (35.09)	76.71 (35.23)	70.81 (37.94)	**<0.001**
**Body pain, points, (SD)**	69.69 (25.25)	65.20 (25.95)	62.46 (26.07)	57.47 (27.48)	**<0.001**
**General health, points, (SD)**	65.74 (17.01)	63.51 (18.67)	61.89 (18.38)	58.62 (19.63)	**<0.001**
**Vitality, points, (SD)**	68.53 (19.95)	65.41 (21.13)	63.51 (21.16)	58.64 (22.57)	**<0.001**
**Social role, points, (SD)**	89.30 (17.88)	87.19 (20.01)	86.22 (21.20)	82.44 (23.54)	**<0.001**
**Emotional role, points, (SD)**	89.74 (27.37)	88.19 (28.58)	87.56 (29.74)	83.67 (33.24)	**<0.001**
**Mental health, points, (SD)**	77.00 (18.10)	76.08 (18.74)	74.62 (19.48)	71.58 (20.75)	**<0.001**
**Physical SF-36 summary, points, (SD)**	47.39 (8.16)	45.51 (8.62)	44.79 (8.67)	42.60 (9.61)	**<0.001**
**Mental SF-36 summary, points, (SD)**	52.11 (9.59)	51.91 (9.86)	51.47 (10.45)	50.23 (11.49)	**<0.001**

HSI, Hepatic steatosis index; SD, Standard deviation; MeDiet, Mediterranean Diet.

Bold for p values < 0.05.

NA, Not applicable.

### Longitudinal Evaluation of NAFLD Status in the Influence of Lifestyle on HRQoL

**Figure 2:**
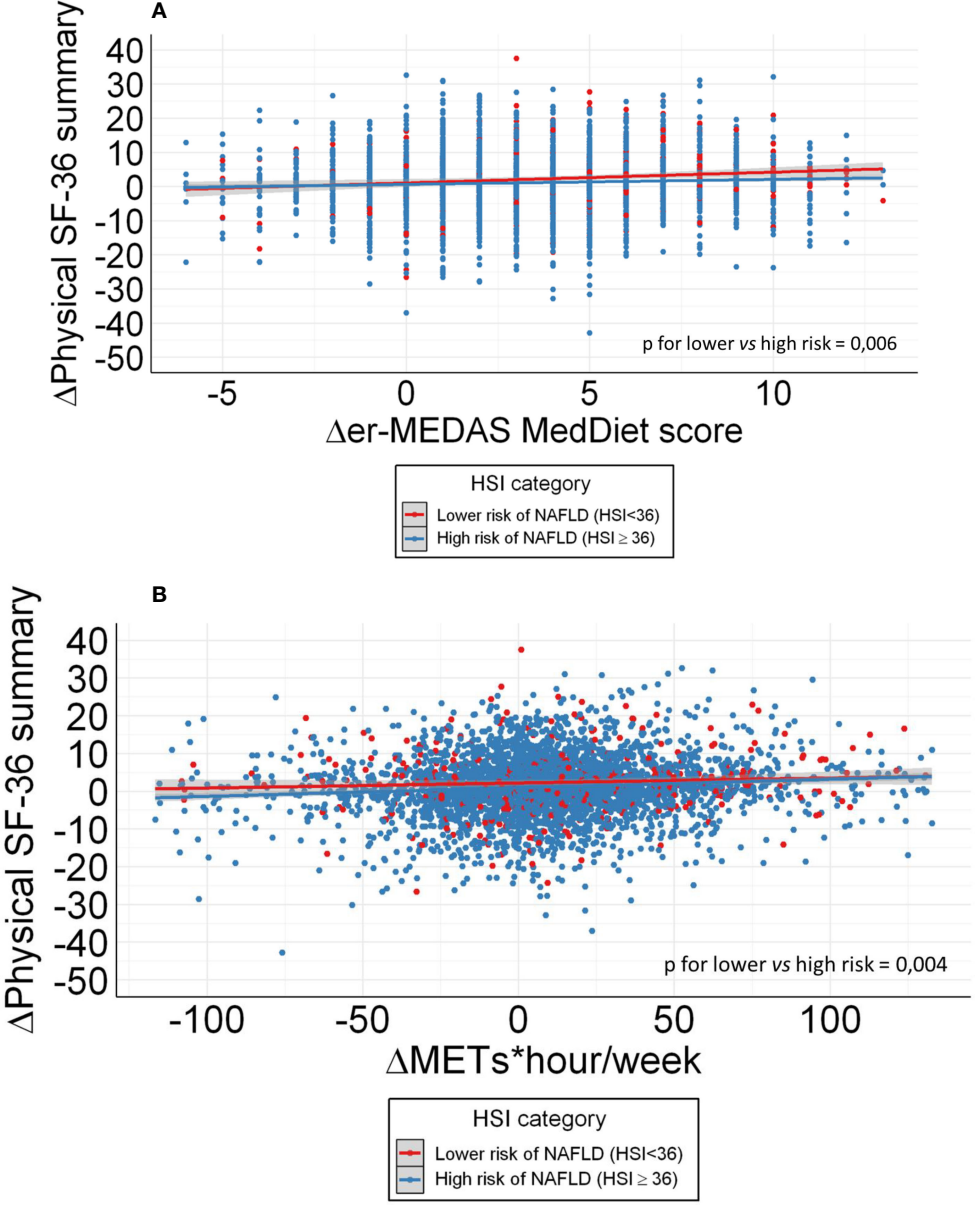
**(A)** Correlation of HRQoL increase and er-MeDiet adherence modification in patients with higher NAFLD probability and lower NAFLD probability. **(B)** Correlation of HRQoL increase and Physical activity modification in patients with higher NAFLD probability and lower NAFLD probability.

For a longitudinal evaluation of the influence of NAFLD in the effect of Lifestyle on HRQoL, a raw correlation analysis was performed featuring the increase in HRQoL and both the er-MeDiet and PA adherence after one year follow-up, dividing patients between those with a HSI score below 36 (lower probability of liver steatosis) and those with 36 points or more in the HSI score (higher probability of liver steatosis). Both models showed a different response in terms of HRQoL to lifestyle changes depending on liver status in both er-MeDiet and PA adherence (p < 0.05 respectively). This data are shown in **Figure 2**.

According to these results, mixed multivariate regression models were developed to evaluate the consequences of er-MeDiet adherence and PA in patients with lower probability of liver steatosis according to HSI < 36 points and those with a high probability of NAFLD (HSI ≥ 36 points) including baseline and 1 year of follow up data.

**Figure 3:**
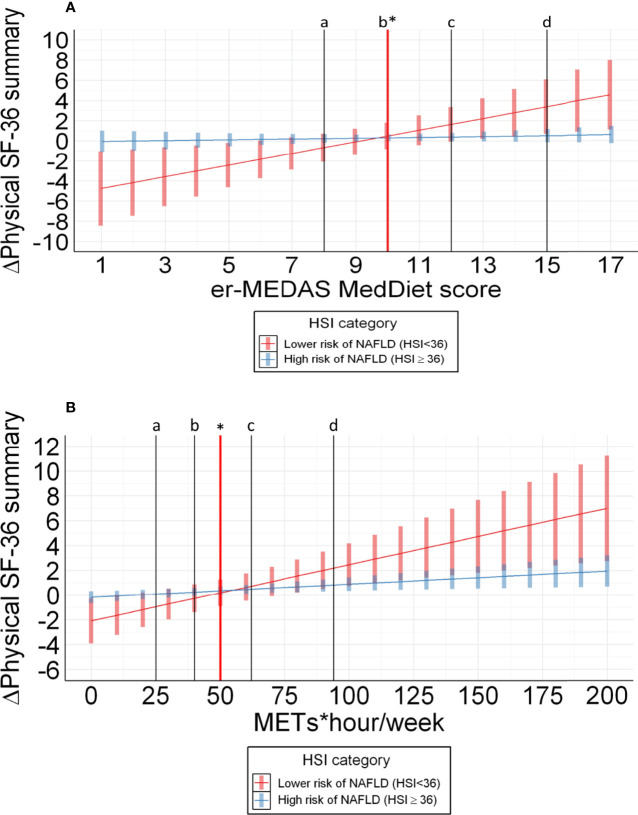
**(A)** Prediction of effect of Mediterranean diet adherence on SF-36 components after 1-year follow up adjusted by age, sex, recruitment node, daily alcohol consumption, total caloric intake, waist circumference and aggregated MetS features. a: percentile 20 for er-MEDAS MedDiet score (8 points) of participants with HSI < 36; b: percentile 40 for er-MEDAS MedDiet score (10 points) of participants with HSI < 36; c: percentile 60 for er-MEDAS MedDiet score (12 points) of participants with HSI < 36; d: percentile 80 for er-MEDAS MedDiet score (15 points) of participants with HSI < 36; * cutoff point (10 points) with the x axis (Δ Physical SF-36 summary = 0) from which patients with HSI < 36 benefit from er-MeDiet adherence in terms of physical HRQoL. **(B)** Prediction of effect of Physical activity on SF-36 components after 1-year follow up adjusted by age, sex, recruitment node, daily alcohol consumption, total caloric intake, hip-waist circumference and aggregated MetS features. a: percentile 20 for METs*hour/week (25 METs*hour/week) of participants with HSI < 36; b: percentile 40 for METs*hour/week (40 METs*hour/week) of participants with HSI ;< 36 c: percentile 60 for METs*hour/week (62 METs*hour/week) of participants with HSI < 36; d: percentile 80 for METs*hour/week (94 METs*hour/week) of participants with HSI < 36; * cutoff point (50 METs*hour/week) with the x axis (Δ Physical SF-36 summary = 0) from which patients with HSI < 36 benefit from PA changes in terms of physical HRQoL.

In the evaluation of er-MeDiet, differences were found between both subgroups in physical role, and PCS after adjustment by age, sex, recruitment node, total calory intake, alcohol consumption, waist circumference, number of items of the MetS and PA. The PCS response to er-MeDiet adherence was found to be higher in patients with lower HSI (β 0.58 CI 95% 0.27-1.00) in comparison with those with a HSI ≥ 36 points (β 0.07 CI 95% -0.06-0.16) as shown (**Table 3A**). These results were not replicated when stratifying patients in the different components of the HSI (ALT/AST ratio, BMI, fasting blood glucose > 100 mg/dl and sex) or by age (**Supplementary Tables 1, 2**). Data were plotted to show the influence of er-MeDiet adherence in terms of HRQoL variation (**Figure 3A**). In this figure, the linear mixed model predicts the annual increase of HRQoL depending on the er-MeDiet score providing that patients with high probability of NAFLD tend to be resistant to increases in er-MeDiet adherence, while patients with lower NAFLD risk are very sensitive to dietary changes in terms of HRQoL aggregated physical domain. Beyond, patients below a 10-point adherence to er-MeDiet, tended to lose HRQoL PCS while those with a score of 10 points or higher, improved HRQoL PCS in the 1-year follow-up (**Figure 3A**).

**Table 3A:** Mixed multivariate model on the effect of Mediterranean diet adherence on SF-36 components after 1-year follow up.

	Indeterminate probability of NAFLD (HSI < 36)			High probability of NAFLDHSI > 36			p for non-NAFLD vs. NAFLD patients
	*β*	*95% Conf. Interval*		*β*	*95% Conf. Interval*		
**Pyisical status, points, (SD)**	0.70	-0.15	1.55	-0.12	-0.35	0.11	0.067
**Physical role, points, (SD)**	2.63	0.77	4.49	0.34	-0.17	0.85	**0.020**
**Body pain, points, (SD)**	1.14	-0.18	2.46	-0.02	-0.37	0.34	0.097
**General health, points, (SD)**	0.96	0.10	1.83	0.44	0.21	0.67	0.255
**Vitality, points, (SD)**	0.46	-0.51	1.42	0.18	-0.08	0.44	0.588
**Social role, points, (SD)**	0.03	-1.05	1.10	0.21	-0.08	0.50	0.744
**Emotional role, points, (SD)**	0.41	-1.20	2.02	0.30	-0.15	0.74	0.893
**Mental health, points, (SD)**	0.22	-0.66	1.11	0.13	-0.11	0.37	0.845
**Physical SF-36 summary, points, (SD)**	0.58	0.16	1.00	0.04	-0.07	0.15	**0.014**
**Mental SF-36 summary, points, (SD)**	-0.11	-0.62	0.41	0.11	-0.03	0.25	0.426

er-MeDiet adherence.

Adjusted by: Age (years), sex, recruitment node, physical activity (METs/h/week), daily alcohol consumption (g/day), total caloric intake (kcal/day), waist circumference and number of Metabolic Syndrome components.

HSI, Hepatic steatosis index; SD, Standard deviation.

Bold for p values < 0.05.

In the evaluation of PA, patients with a higher improvement in physical role, body pain and vitality scores in response to increases in PA were associated to a lower HSI after previously described adjustments (**Table 3B**). In the evaluation of PA, the HSI influence on HRQoL evolution was not replicated when stratifying patients in the different components of the HSI (ALT/AST ratio, BMI, diabetes and sex) or by age (**Supplementary Tables 2, 3**). The PCS response to PA was found to improve more in patients with lower HSI (β 0.05 CI 95% 0.03-0.07) in comparison with those with a HSI ≥ 36 points (β 0.03 CI 95% 0.01-0.02) as described (**Table 3B** and **Figure 3B**). Besides, patients performing less than 50 MET-h/week of PA, tended to lose HRQoL while those accomplishing more PA, improved HRQoL in the 1-year follow-up (**Figure 3B**). Additional information for the interpretation of this results about the predicted PCS year increase depending on different levels of PA and er-MeDiet are also provided (**Supplementary Figure 2**). After this analysis, the regression model was replicated among quartiles of HSI to provide information about progressive response of HRQoL depending on lifestyle. Although no important statistically significant results were found in this approach, a trend towards statistical significance was found in the Physical Activity adherence group (**Supplementary Tables 4, 5**).

**Table 3B:** Physical activity performance.

	Lower probability of NAFLD (HSI < 36)			Higher probability of NAFLDHSI > 36			p for non-NAFLD vs. NAFLD patients
	*β*	*95% Conf. Interval*		*β*	*95% Conf. Interval*		
**Pyisical status, points, (SD)**	0.030	-0.027	0.086	0.015	-0.001	0.031	0.619
**Physical role, points, (SD)**	0.179	0.054	0.304	0.034	-0.002	0.070	**0.030**
**Body pain, points, (SD)**	0.123	0.035	0.211	0.021	-0.004	0.046	**0.031**
**General health, points, (SD)**	0.012	-0.046	0.069	0.015	-0.001	0.031	0.907
**Vitality, points, (SD)**	0.050	-0.014	0.114	-0.003	-0.021	0.015	0.126
**Social role, points, (SD)**	-0.001	-0.073	0.071	0.007	-0.014	0.028	0.845
**Emotional role, points, (SD)**	-0.035	-0.144	0.073	-0.0004	-0.032	0.031	0.545
**Mental health, points, (SD)**	-0.033	-0.092	0.026	-0.002	-0.018	0.015	0.323
**Physical SF-36 summary, points, (SD)**	0.046	0.018	0.073	0.011	0.003	0.019	**0.019**
**Mental SF-36 summary, points, (SD)**	-0.026	-0.061	0.008	-0.004	-0.014	0.005	0.229

Adjusted by: Age (years), sex, recruitment node, Mediterranean diet score, daily alcohol consumption (g/day), total caloric intake (kcal/day), waist circumference and number of Metabolic Syndrome components.

HSI, Hepatic steatosis index; SD, Standard deviation.

Bold for p values < 0.05.

## Discussion

The results from the current research evidence the capacity of a non-invasive evaluation of NAFLD to modify the effect of lifestyle over Health-related Quality of Life. The increase in the adherence to a er-MedDiet pattern and PA might lead to more than a 5-fold increase in the aggregated physical domain of HRQoL depending on the presence of NAFLD, using previously validated and easy to fulfill scores such as SF-36 and HSI (18, 20). In this context, different methods of detection of liver steatosis have been previously validated, such as ultrasound, controlled attenuation parameters of FibroScan^©^. Among them, HSI has demonstrated adequacy in the epidemiological study of liver disease (21), while demonstrating a high similar discrimination power than other more expensive or less available detection methods (22). Besides, our results provide a clinically relevant objectives in terms of er-MeDiet adherence and PA to improve HRQoL in patients with MetS and lower probability of NAFLD. To our knowledge, this is the first investigation to proof the differential effect of lifestyle measures in patients with MetS depending on a non-invasive assessment of liver steatosis.

Interestingly, in our cohort, the HSI score was associated to a lower adherence to er-MeDiet and PA performance and to a higher prevalence and of the components of MetS at baseline. These findings are endorsed by indirect findings and provide further evidence on the association of HSI score and the MetS features (3). Furthermore, these results reinforce the impact of liver steatosis in QoL (20) and might support previous evidence on the use of non-invasive methods for the individualization of patients with MetS in the clinical setting (23–25). Thus, the apparent resistance of patients with NAFLD to lifestyle measures in terms of HRQoL in the longitudinal analysis could adequate the prescription of the therapeutic armamentarium in these patients, bringing forward more aggressive treatments in selected subjects, such as GLP-1 analogs and bariatric surgery (26, 27). Beyond, the 10 point er-MeDiet adherence and 50 MET-h/week thresholds for the improvement of HRQoL in patients with lower probability of NAFLD may provide a goal of lifestyle change in these patients as well as an objective evaluation and counselling in the clinical setting. Therefore, our study could contribute to the development of new lifestyle-based intervention guidelines for patients with MetS according to the liver status which in fact, may also provide further benefits in the cardiovascular disease scenario, due to the association of NAFLD and atherosclerotic disease development (28, 29).

With regard of the impact of Mediterranean diet and PA in HRQoL depending on the liver status, some aspects should be emphasized. The effect of Mediterranean diet has been previously related to the MetS prevention and control after 1 year follow up (30). Besides, MedDiet pattern has also been related to HRQoL in previous series and in the present cohort (31). Similar results were found on PA relationship to HRQoL, with a direct relationship to the control of MetS and HRQoL. However, the concept of a modulation of NAFLD status in the impact of er-MeDiet and PA on HRQoL is new. In fact, interventions tend to be maximized in patients at higher risk (32). Thus, the capacity of HSI to identify patients with a better response to lifestyle as measured with HRQoL parameters might be related to the NAFLD morbidity burden (33). Other factor that could contribute to our results is the severity of the MetS among progressive HSI values. This fact might be related to a poorer metabolic flexibility of patients with NAFLD in terms of body composition (34), insulin resistance (35), microbioma (9) and redox/equilibrium (36) and thus, a reduction on the sensitivity to lifestyle change of this population due to a reduction in homeostasis capability (37).

In the methodological arena, the design quality of PREDIMED plus provides strength to the present results in terms of inclusion criteria fulfillment, anthropometric and laboratory tests and adherence to er-MeDiet and PA patterns assessment (19). Besides, the SF-36 Physical summary and the HSI index are well validated and universally used indexes in the evaluation of both HRQoL and liver steatosis (18, 20). Even though, some limitations should be addressed. First, the cohort recruitment was designed to evaluate the effect of the intervention on dietary patterns and PA in patients with MetS. Then, the present findings should be externally validated in a different cohort. In this context, the present analysis does not follow the original strategy of the cohort, resembling to a per-protocol evaluation by using patient information about erMeDiet and PA adherence according to patient declaration and not due to the distribution between the control and intervention branches. Although the characteristics of the full PREDIMED plus trial do not allow to uncover the intervention and control groups, no differences were found in the proportion of patients in the intervention and control groups among HSI < 36 and HSI > 36 (p > 0.05). Besides, although HSI is a remarkable non-invasive tool in the detection of NAFLD, this index is not designed for NAFLD staging. In fact, biopsy directed studies have demonstrated a different distribution of NASH in patients with MetS than in those with type 2 diabetes mellitus (38). Although the NAFLD staging may exceed the objective of the present study, this feature could be addressed in future studies. Yet, the present results are valuable from the clinical point of view, providing remarkably similar assessment of the influence of adherence to healthy lifestyle patterns in patients’ health as that of the consultation room. The longitudinal multivariate analysis and adjustments, using features related to HRQoL such as waist circumference obesity, MetS features (39), alcohol consumption (40) and total caloric intake (41, 42) provide plausibility to the present results.

In summary, the demonstration of the influence of NAFLD in the response to lifestyle modifications might represent an interesting tool for personalization and precision medicine in the cardiovascular risk epidemiological and clinical scenarios emphasizing the mediation of liver status in the therapeutical response. The present findings could encourage future research in the individualization of treatment of patients with MetS based on liver condition.

## Conclusion

The detection of NAFLD by the HSI index in patients with MetS might identify subjects with lower sensitivity to lifestyle changes in terms of HRQoL. Patients with lower NAFLD probability begun to obtain improvement on HRQoL at 10 points of er-MeDiet adherence and 50 MET-h/week, providing precision medicine prescriptions depending on liver condition status.

## Data Availability Statement

There are restrictions on data availability for the PREDIMED-Plus trial due to the signed consent agreements around data sharing, which only allow access to external researchers for studies following the project purposes. Requestors wishing to access the PREDIMED-Plus trial data used in this study can make a request to the PREDIMED-Plus trial Steering Committee chair: jordi.salas@urv.cat. The request will then be passed to members of the PREDIMED-Plus Steering Committee for deliberation.

## Ethics Statement

The studies involving human participants were reviewed and approved by Predimed Plus trial. The patients/participants provided their written informed consent to participate in this study.

## Author Contributions

MAM-G, NB, DC, JLV, AMA-G, JW, JesV, DR, JL-M, RE, FT, JL, JS-M, AB-C, JT, AM, XP, MD-R, PMM, JosV, MM, IA, AM-R, IC-G, JK, AG-R, OL, OP, AG, MZ, JV-L, MCSO, IM, EB, JAM, JO and LD designed and conducted the research. DM-U, RS-C, PV, JM and LD conceived the study idea and the analysis design. LD supervised the research and DM-U and RS-C carried out the analysis procedures, bibliographic research, data preparation, statistical analysis and wrote initial drafts. RS-C assisted with statistical analysis and R programming. PV and JO participated in the scientific discussion of experimental results. All the authors assisted in manuscript revision for intellectual content and approved it.

## Funding

The present study received funding from coordinated FIS project official Spanish institutions for funding scientific biomedical research, CIBER Fisiopatología de la Obesidad y Nutrición (CIBEROBN) and Instituto de Salud Carlos III (ISCIII) through the Fondo de Investigación para la Salud (FIS) which is co-funded by the European Regional Development Fund (including the following projects: PI13/00673, PI13/00492, PI13/00272, PI13/01123, PI13/00462, PI13/00233, PI13/02184, PI13/00728, PI13/01090, PI13/01056, PI14/01722, PI14/00636, PI14/00618, PI14/00696, PI14/01206, PI14/01919, PI14/00853, PI14/01374, PI14/00972, PI14/00728, PI14/01471, PI16/00473, PI16/00662, PI16/01873, PI16/01094, PI16/00501, PI16/00533, PI16/00381, PI16/00366, PI16/01522, PI16/01120, PI17/00764, PI17/01183, PI17/00855, PI17/01347, PI17/00525, PI17/01827, PI17/00532, PI17/00215, PI17/01441, PI17/00508, PI17/01732, PI17/00926, PI19/00957, PI19/00386, PI19/00309, PI19/01032, PI19/00576, PI19/00017, PI19/01226, PI19/00781, PI19/01560, PI19/01332, PI20/01802, PI20/00138, PI20/01532, PI20/00456, PI20/00339, PI20/00557, PI20/00886, PI20/01158), the Special Action Project “Implementación y evaluación de una intervención intensiva sobre la actividad física Cohorte PREDIMED-Plus”, the Recercaixa (grant number 2013ACUP00194) funding and the grants from the Consejería de Salud de la Junta de Andalucía (PI0458/2013; PS0358/2016; PI0137/2018), the SEMERGEN grant; Department of Health of the Government of Navarra (61/2015), the Fundació La Marató de TV (Ref. 201630.10), the coordinated grant supported by the European Research Council (Advanced Research grant 2014–2019; agreement #340918), the grant of support to research groups 35/2011 (Balearic Islands Government; FEDER funds). This study has been carried out thanks to the funds provided by Ministerio de Ciencia e Investigación through grants from PROYECTOS DE I+D+I «RETOS INVESTIGACIÓN» (RTI2018-095569-B-I00) and «PROGRAMACIÓN CONJUNTA INTERNACIONAL» (PCI2018-093009).

## Conflict of Interest

Author JS-S reports grants from CIBEROBN, ISCIII (Spain), during the conduct of the study; non-financial support from Nut and Dried Fruit Foundation, personal fees from Instituto Danone Spain, grants from Nut and Dried Fruit Foundation to this institution, grants from Eroski Distributors and personal fees from Nut and Dried Fruit Foundation, outside the submitted work. Moreover, he gratefully acknowledges the financial support by ICREA under the ICREA Academia program. The author DR declares the funding of the AstraZeneca Young Investigators Award in Category of Obesity and T2D 2017. The author RS-C acknowledges financial support from the Juan de la Cierva Program Training Grants of the Spanish State Research Agency of the Spanish Ministerio de Ciencia e Innovación y Ministerio de Universidades (FJC2018-038168- I). The author ER reports grants, personal fees, non-financial support and other from California Walnut Commission, grants, personal fees, non-financial support and other from Alexion, personal fees, non-financial support and other from Ferrer International, personal fees from Amarin, personal fees, non-financial support and other from Danone, outside the submitted work. The author JL-M reports personal fees and non-financial support from AMGEN, personal fees and non-financial support from SANOFI, personal fees from MSD, personal fees from Laboratorios Dr. Esteve, and personal fees from NOVO-NORDISK, outside the submitted work.

The remaining authors declare that the research was conducted in the absence of any commercial or financial relationships that could be construed as a potential conflict of interest.

## Publisher’s Note

All claims expressed in this article are solely those of the authors and do not necessarily represent those of their affiliated organizations, or those of the publisher, the editors and the reviewers. Any product that may be evaluated in this article, or claim that may be made by its manufacturer, is not guaranteed or endorsed by the publisher.
