# Corrigendum: Role of NAFLD on the health related QoL response to lifestyle in patients with metabolic syndrome: The PREDIMED plus cohort

**DOI:** 10.3389/fendo.2022.1113532

**Published:** 2023-01-11

**Authors:** Diego Martínez-Urbistondo, Rodrigo San-Cristóbal, Paula Villares, Miguel Ángel Martínez-González, Nancy Babio, Dolores Corella, José Luis del Val, José Ma Ordovás, Ángel M. Alonso-Gómez, Julia Wärnberg, Jesús Vioque, Dora Romaguera, José López-Miranda, Ramon Estruch, Francisco J. Tinahones, José Lapetra, J. Luís Serra-Majem, Aurora Bueno-Cavanillas, Josep A. Tur, Alba Marcos, Xavier Pintó, Miguel Delgado-Rodríguez, Pilar Matía-Martín, Josep Vidal, Clotilde Vázquez, Emilio Ros, María Vanessa Bullón Vela, Antoni Palau, Jose V. Sorli, Marta Masagué, Itziar Abete, Anai Moreno-Rodríguez, Inma Candela-García, Jadwiga Konieczna, Antonio García-Ríos, Oscar Lecea Juárez, Olga Portolés, Paco Martín, Albert Goday, M. Ángeles Zulet, Jessica Vaquero-Luna, María del Carmen Sayón Orea, Isabel Megías, Enric Baltasar, J. Alfredo Martínez, Lidia Daimiel

**Affiliations:** ^1^ Internal Medicine Department, Hospital HM Sanchinarro, HM Hospitales, Madrid, Spain; ^2^ Cardiometabolic Nutrition Group, Precision Nutrition and Cardiometabolic Health Program, Instituto Madrileño de Estudios Avanzados (IMDEA) Food, Centro de Excelencia en Investigación (CEI) Universidad Autónoma de Madrid (UAM) + Centro Superior de Investigaciones Científicas (CSIC), Madrid, Spain; ^3^ Department of Preventive Medicine and Public Health, University of Navarra, IdiSNA, Pamplona, Spain; ^4^ Consorcio CIBER, M.P. Fisiopatología de la Obesidad y Nutrición (CIBERObn), Instituto de Salud Carlos III (ISCIII)., Madrid, Spain; ^5^ Department of Nutrition, Harvard T. H. Chan School of Public Health, Boston, MA, United States; ^6^ Universitat Rovira i Virgili, Departament de Bioquímica i biotecnologia, Unitat de Nutrició Humana, Reus, Spain; ^7^ Institut d’Investigació Sanitària Pere Virgili (IISPV), Hospital Universitari San Joan de Reus. Human Nutrition unit, Reus, Spain; ^8^ Department of Preventive Medicine, University of Valencia, Valencia, Spain; ^9^ Cardiovascular Risk and Nutrition Research Group (CARIN), Hospital del Mar Medical Research Institute (IMIM), Barcelona, Spain; ^10^ Nutritional Genomics and Epigenomics Group, Precision Nutrition and Obesity Program. Instituto Madrileño de Estudios Avanzados (IMDEA) Food, Centro de Excelencia en Investigación (CEI) Universidad Autónoma de Madrid (UAM) + Centro Superior de Investigaciones Científicas (CSIC), Madrid, Spain; ^11^ Nutrition and Genomics Laboratory, Jean Mayer United States Department of Agriculture (JM_USDA) Human Nutrition Research Center on Aging, Tufts University, Boston, MA, United States; ^12^ Bioaraba Health Research Institute, Osakidetza Basque Health Service, Araba University Hospital, University of the Basque Country Universidad del País Vasco/Euskal Herriko Unibertsitatea (UPV/EHU), Vitoria-Gasteiz, Spain; ^13^ Department of Nursing, School of Health Sciences, University of Málaga, Instituto de Investigación Biomédica de Málaga (IBIMA), Málaga, Spain; ^14^ Centro de Investigación Biomédica en Red de Epidemiología y Salud Pública (CIBERESP), Instituto de Salud Carlos III, Madrid, Spain; ^15^ Instituto de Investigación Sanitaria y Biomédica de Alicante. Universidad Miguel Hernández (ISABIAL-UMH)., Alicante, Spain; ^16^ Research Group on Nutritional Epidemiology & Cardiovascular Physiopathology (NUTRECOR). Health Research Institute of the Balearic Islands (IdISBa), University Hospital Son Espases (HUSE), Palma de Mallorca, Spain; ^17^ Lipids and Atherosclerosis Unit, Department of Internal Medicine, Maimonides Biomedical Research Institute of Cordoba (IMIBIC), Reina Sofia University Hospital, University of Cordoba, Córdoba, Spain; ^18^ Department of Internal Medicine, Instituto de Investigaciones Biomédicas August Pi i Sunyer (IDIBAPS), Hospital Clinic, University of Barcelona, Barcelona, Spain; ^19^ Department of Endocrinology, Instituto de Investigación Biomédica de Málaga (IBIMA), Virgen de la Victoria Hospital, University of Málaga, Málaga, Spain; ^20^ Department of Family Medicine, Research Unit, Distrito Sanitario Atención Primaria Sevilla, Sevilla, Spain; ^21^ Research Institute of Biomedical and Health Sciences Instituto Universitario de Investigaciones Biomédicas y Sanitarias (IUIBS), University of Las Palmas de Gran Canaria, Preventive Medicine Service, Centro Hospitalario Universitario Insular Materno Infantil (CHUIMI), Canarian Health Service, Las Palmas, Spain; ^22^ Department of Preventive Medicine and Public Health, University of Granada, Granada, Spain; ^23^ Research Group on Community Nutrition & Oxidative Stress, University of Balearic Islands, Palma de Mallorca, Spain; ^24^ Institute of Biomedicine (IBIOMED), University of León, León, Spain; ^25^ Lipids and Vascular Risk Unit, Internal Medicine, Hospital Universitario de Bellvitge, Hospitalet de Llobregat, Barcelona, Spain; ^26^ Departamento de Ciencias de la Salud, Centro de Estudios Avanzados en Olivar y Aceites de Oliva, Universidad de Jaén, Jaén, Spain; ^27^ Department of Endocrinology and Nutrition, Instituto de Investigación Sanitaria Hospital Clínico San Carlos (IdISSC), Madrid, Spain; ^28^ Biomedical Research Centre for Diabetes and Metabolic Diseases Network (CIBERDEM), Instituto de Salud Carlos III (ISCIII), Madrid, Spain; ^29^ Endocrinology and Nutrition Service, Instituto de Investigaciones Biomédicas August Pi i Sunyer (IDIBAPS), Hospital Clinic, University of Barcelona, Barcelona, Spain; ^30^ Department of Endocrinology and Nutrition, Hospital Fundación Jimenez Díaz, Instituto de Investigaciones Biomédicas IISFJD. University Autónoma, Madrid, Spain; ^31^ Department of Nutrition, Food Sciences and Physiology, University of Navarra, Pamplona, Spain; ^32^ Centro de Salud San Pola, Alicante, Spain; ^33^ Departament de Medicina, Universitat Autònoma de Barcelona, Barcelona, Spain; ^34^ Nutritional Control of the Epigenome Group. Precision Nutrition and Obesity Program. Instituto Madrileño de Estudios Avanzados (IMDEA) Food, Centro de Excelencia en Investigación (CEI) Universidad Autónoma de Madrid (UAM) + Centro Superior de Investigaciones Científicas (CSIC), Madrid, Spain

**Keywords:** NAFLD, metabolic syndrome, mediterranean diet, physical activity, HRQoL

**Incorrect Author Name**

In the published article, an author name was incorrectly written as Rodrigo San Cristobal. The correct spelling is Rodrigo San-Cristobal.

The authors apologize for this error and state that this does not change the scientific conclusions of the article in any way. The original article has been updated.

**Error in Author List**

In the published article, there was an error in the author list, and authors Olga Portoles and Jose V Sorli were erroneously excluded. The corrected author list appears below.

Diego Martínez-Urbistondo^1^*, Rodrigo San-Cristóbal^2^, Paula Villares^1^, Miguel Ángel Martínez-González^3,4,5^, Nancy Babio^4,6,7^, Dolores Corella^4,8^, José Luis del Val^4,9^, José M^a^ Ordovás^10,11^, Ángel M. Alonso-Gómez^4,12^, Julia Wärnberg^4,13^, Jesús Vioque^14,15^, Dora Romaguera^4,16^, José López-Miranda^4,17^, Ramon Estruch^4,18^, Francisco J Tinahones^4,19^, José Lapetra^4,20^, J. Luís Serra-Majem^4,21^, Aurora Bueno-Cavanillas^14,22^, Josep A. Tur^4,23^, Alba Marcos^14,24^, Xavier Pintó^4,25^, Miguel Delgado-Rodríguez^14,26^, Pilar Matía-Martín^27^, Josep Vidal^28,29^, Clotilde Vázquez^4,30^, Emilio Ros^4,29^, María Vanessa Bullón Vela^3,4^, Antoni Palau^4,6,7^, Jose V Sorli^4,8^, Marta Masagué^4,9^, Itziar Abete^4,33^, Anai Moreno-Rodríguez^4,12^, Inma Candela-García^31^, Jadwiga Konieczna^4,16^, Antonio García-Ríos^4,17^, Oscar Lecea Juárez^3,4^, Olga Portolés^4,8^, Paco Martín^4,6,7^, Albert Goday^4,9,32^, M Ángeles Zulet^4,33^, Jessica Vaquero-Luna^4,12^, María del Carmen Sayón Orea^3,4^, Isabel Megías^4,6,7^, Enric Baltasar^4,9^, J. Alfredo Martínez^2,4,33^, Lidia Daimiel^34^

The Author Contributions section should include these authors and has been updated as follows:

## Author contributions

MAM-G, NB, DC, JLV, AMA-G, JW, JesV, DR, JL-M, RE, FT, JL, JS-M, AB-C, JT, AM, XP, MD-R, PM-M, JosV, MM, IA, AM-R, IC-G, JK, AG-R, OL, OP, AG, MZ, JV-L, MCSO, IM, EB, JAM, JO and LD designed and conducted the research. DM-U, RS-C, PV, JM and LD conceived the study idea and the analysis design. LD supervised the research and DM-U and RS-C carried out the analysis procedures, bibliographic research, data preparation, statistical analysis and wrote initial drafts. RS-C assisted with statistical analysis and R programming. PV and JO participated in the scientific discussion of experimental results. All the authors assisted in manuscript revision for intellectual content and approved it.

The authors apologize for this error and state that this does not change the scientific conclusions of the article in any way. The original article has been updated.

