# Supplementary material for: Role of NAFLD on the Health Related QoL Response to Lifestyle in Patients With Metabolic Syndrome: The PREDIMED Plus Cohort
Source: Front Endocrinol (Lausanne). 2022 Jun 29;13:868795. doi: 10.3389/fendo.2022.868795 (PMC9276971; doi:10.3389/fendo.2022.868795)
Supplement: Supplementary file 1 [file DataSheet_1.docx]

**Appendix 1**

Predimed is an ongoing, 6-year randomized, controlled, parallel-group clinical trial for primary prevention of CVD through lifestyle modification in Spanish patients. The standardized operation procedures and cohort features, including sample size estimation and enrollment flowchart, have been described elsewhere, and the protocol can be found at the PREDIMED-Plus webpage. This trial was retrospectively registered at the International Standard Randomized Controlled Trial directory (ISRCTN89898870,https://www.isrctn.com/SRCTN89898870?q=ISRCTN89898870&flters=&sort=&ofset=1&totalResults=1&page=1&pageSize=10& searchType=basic-search) on 07/24/2014.

Volunteer participants were enrolled by primary care health doctors and nurses associated with the involved study centers, which supervised the fulfillment of the inclusion and exclusion criteria. Qualified participants were community-dwelling adults (55–75 years men and 60–75 years women) with overweight or obesity (27 ≤ BMI ≥ 40 Kg/m^2^) who met at least three MetS requirements according to the International Diabetes Federation and the American Heart Association with no cardiovascular and neurodegenerative disease diagnosis at baseline. Baseline data from 6874 recruited participants in 23 Spanish study centers were included from October 2013 to December 2016. Data encompassed demographic, co-morbidity, lifestyle and quality of life data, as well as anthropometrical and biochemical measures, at baseline and after 1 year of follow up were used. The study protocol and procedures were approved according to the ethical standards of the Declaration of Helsinki by the Institutional Review Boards of all the participating institutions. All participants provided informed written consent.

*Dietary and anthropometrical measures*

Adherence to a specifically Energy restricted Mediterranean Diet (er-MedDiet) was measured through a 17-item questionnaire, where each adequate intake of traditional Mediterranean foods summed one point while a low consumption of those foods that are not common of the traditional Mediterranean diet also increased one point. This survey was specially devised for the PREDIMED-Plus trial and was questioned by a trained dietitian/nutritionist within a face-to-face interview. Weight and height determinations were assessed in light clothing and no shoes, with previously calibrated tools and a wall-mounted stadiometer, respectively. BMI was calculated as weight in kilograms divided by the square of height in meters.

*Physical activity*

PA was assessed using the validated REGICOR questionnaire and the Rapid Assessment of Physical Activity Questionnaire (RAPA). REGICOR questionnaire provides information about PA intensity according to energy expenditure (MET/min/week) distinguishing light (< 4 MET), moderate (4-5.5 MET) and vigorous (≥ 6 MET) PA and recording information about the PA at work and in everyday life and about sedentary behaviors^29^. Frequency and intensity of PA are quantified by requesting participants to provide information about the frequency (days per month) and the intensity (minutes per session) of physical activities like light walking, brisk walking, gardening, trail/hiking, climbing stairs, other indoor or outdoor sports activities. RAPA questionnaire allows an easy identification of PA level as low (light-moderate PA activities but not every week), moderate (moderate PA activities less than 150 minutes per week or vigorous PA activities less than 75 minutes per week) or high (more than 150 minutes per week of moderate PA activities or more than 75 minutes per week of vigorous activities). Trained interviewers administered PA questionnaires in individual face-to-face sessions.

*References*

Martínez-González MA, Buil-Cosiales P, Corella D, Bulló M, Fitó M, Vioque J, Romaguera D, Martínez JA, Wärnberg J, López-Miranda J, Estruch R, Bueno-Cavanillas A, Arós F, Tur JA, Tinahones F, Serra-Majem L, Martín V, Lapetra J, Vázquez C, Pintó X, Vidal J, Daimiel L, Delgado-Rodríguez M, Matía P, Ros E, Fernández-Aranda F, Botella C, Portillo MP, Lamuela-Raventós RM, Marcos A, Sáez G, Gómez-Gracia E, Ruiz-Canela M, Toledo E, Alvarez-Alvarez I, Díez-Espino J, Sorlí JV, Basora J, Castañer O, Schröder H, Navarrete-Muñoz EM, Zulet MA, García-Rios A, Salas-Salvadó J; PREDIMED-Plus Study Investigators. Cohort Profile: Design and methods of the PREDIMED-Plus randomized trial. Int J Epidemiol. 2019 Apr 1;48(2):387-388o. doi: 10.1093/ije/dyy225. PMID: 30476123.

Alberti KG, Eckel RH, Grundy SM, Zimmet PZ, Cleeman JI, Donato KA, Fruchart JC, James WP, Loria CM, Smith SC Jr; International Diabetes Federation Task Force on Epidemiology and Prevention; Hational Heart, Lung, and Blood Institute; American Heart Association; World Heart Federation; International Atherosclerosis Society; International Association for the Study of Obesity. Harmonizing the metabolic syndrome: a joint interim statement of the International Diabetes Federation Task Force on Epidemiology and Prevention; National Heart, Lung, and Blood Institute; American Heart Association; World Heart Federation; International Atherosclerosis Society; and International Association for the Study of Obesity. Circulation. 2009 Oct 20;120(16):1640-5. doi: 10.1161/CIRCULATIONAHA.109.192644. Epub 2009 Oct 5. PMID: 19805654.

Schröder H, Zomeño MD, Martínez-González MA, Salas-Salvadó J, Corella D, Vioque J, Romaguera D, Martínez JA, Tinahones FJ, Miranda JL, Estruch R, Bueno-Cavanillas A, Alonso Gómez AM, Tur JA, Warnberg J, Serra-Majem L, Martín V, Vázquez C, Lapetra J, Pintó X, Vidal J, Daimiel L, Gaforio JJ, Matía-Martín P, Ros E, Lassale C, Ruiz-Canela M, Babio N, Sorlí JV, García-Arellano A, Díaz-López A, Fitó M, Castañer O; PREDIMED-Plus investigators. Validity of the energy-restricted Mediterranean Diet Adherence Screener. Clin Nutr. 2021 Jul 6;40(8):4971-4979. doi: 10.1016/j.clnu.2021.06.030. Epub ahead of print. PMID: 34364236..

Molina L, Sarmiento M, Peñafiel J, Donaire D, Garcia-Aymerich J, Gomez M, Ble M, Ruiz S, Frances A, Schröder H, Marrugat J, Elosua R. Validation of the Regicor Short Physical Activity Questionnaire for the Adult Population. PLoS One. 2017 Jan 13;12(1):e0168148. doi: 10.1371/journal.pone.0168148. PMID: 28085886; PMCID: PMC5234797.

**Supplementary tables**

**Suppl Table 1. Population characteristics**

|  | **HSI <36** | **HSI >36** | **p for t-test** |
| --- | --- | --- | --- |
| **n** | 244 | 4777 |  |
| **Age, years (SD)** | 65.9 (5.1) | 65.1 (4.9) | **0.007** |
| **Sex, female (%)** | 65 (26.6) | 2352 (49.2) | **<0.001** |
| **Civil status (%)** |  |  | 0.318 |
| **Single** | 8 (3.3) | 240 (5.0) |  |
| **Married** | 186 (76.2) | 3671 (77.0) |  |
| **Widowed/divorced** | 50 (20.5) | 856 (18.0) |  |
| **Education level (%)** |  |  | 0.164 |
| **Primary** | 107 (43.9) | 2372 (49.7) |  |
| **Secondary** | 75 (30.7) | 1386 (29.0) |  |
| **College** | 62 (25.4) | 1019 (21.3) |  |
| **Working status, inactive (%)** | 193 (79.1) | 3812 (80.1) | 0.764 |
| **Body mass index, kg/m^2^, (SD)** | 28.1 (1.1) | 32.7 (3.4) | **<0.001** |
| **Waist circumference, cm, (SD)** | 99.6 (6.3) | 107.9 (9.5) | **<0.001** |
| **Adherence to MeDiet, 0-17, (SD)** | 8.3 (2.7) | 8.5 (2.6) | 0.398 |
| **Physical activity, METs-min/week, (SD)** | 2992 (2451) | 2495 (2314) | **0.001** |
| **Sedentarism, Yes (%)** | 94 (38.5) | 2099 (44.0) | 0.109 |
| **Energy intake reported, Kcal/day, (SD)** | 2429 (557) | 2348 (552) | **0.025** |
| **Alcohol intake, g/day, (SD)** | 13.8 (16.6) | 10.8 (14.8) | **0.002** |
| **Fasting blood glucosa, mg/dl, (SD)** | 101.1 (17.9) | 114.1 (27.6) | **<0.001** |
| **Fasting blood tryglicerides, mg/dl, (SD)** | 132.3 (53.6) | 141.3 (52.9) | **0.009** |
| **Fasting High-density lipoprotein, mg/dl, (SD)** | 48.5 (12.2) | 48.0 (11.6) | 0.532 |
| **Alanin aminotransferase, UI/L, (SD)** | 17.3 (6.4) | 27.2 (15.2) | **<0.001** |
| **Aspartate aminotransferase, UI/L, (SD)** | 23.6 (10.2) | 23.1 (9.5) | 0.434 |
| **Hepatic steatosis index, points (SD)** | 34.8 (1.0) | 43.7 (5.6) | **<0.001** |
| **Systolic blood pressure (mmHg)** | 137.5 (15.8) | 139.8 (16.9) | **0.04** |
| **Diastolic blood pressure (mmHg)** | 80.3 (9.2) | 80.7 (9.9) | 0.59 |
| **Diagnosed hypertension** | 203 (84.2) | 3984 (84.0) | 0.994 |
| **Diagnosed diabetes** | 15 (6.1) | 1343 (28.2) | **<0.001** |

**Supplementary table 2: Contrast of differences between Indeterminate probability of NAFLD (HSI < 36) and High probability of NAFLD (HSI > 36) of mixed multivariate model on the effect of Mediterranean diet adherence on SF-36 components after 1-year follow up.**

**Suppl. Table 2a: stratified by age group:**

|  | **Age ≤65years n = 2002** | | | **Age >65years n = 1900** | | | **p for contrast** |
| --- | --- | --- | --- | --- | --- | --- | --- |
|  | *β* | *95% Conf. Interval* | | *β* | *95% Conf. Interval* | |  |
| **Physical status, points, (SD)** | -0.53 | -1.83 | 0.78 | -1.07 | -2.26 | 0.13 | 0.55 |
| **Physical role, points, (SD)** | -3.11 | -5.99 | -0.23 | -1.64 | -4.26 | 0.99 | 0.458 |
| **Body pain, points, (SD)** | -1.42 | -3.46 | 0.61 | -0.63 | -2.49 | 1.24 | 0.57 |
| **General health, points, (SD)** | -0.35 | -1.68 | 0.98 | -0.38 | -1.60 | 0.84 | 0.971 |
| **Vitality, points, (SD)** | 0.23 | -1.25 | 1.72 | -0.53 | -1.89 | 0.84 | 0.46 |
| **Social role, points, (SD)** | -0.46 | -2.11 | 1.20 | 0.87 | -0.64 | 2.38 | 0.245 |
| **Emotional role, points, (SD)** | -1.17 | -3.66 | 1.33 | 0.84 | -1.43 | 3.11 | 0.244 |
| **Mental health, points, (SD)** | -0.19 | -1.56 | 1.17 | 0.17 | -1.08 | 1.42 | 0.699 |
| **Physical SF-36 summary, points, (SD)** | -0.47 | -1.11 | 0.17 | -0.56 | -1.15 | 0.03 | 0.845 |
| **Mental SF-36 summary, points, (SD)** | -0.01 | -0.81 | 0.78 | 0.49 | -0.24 | 1.21 | 0.363 |

**Suppl. Table 2b: stratified by obesity prevalence:**

|  | **Overweight (BMI < 30) n = 1085** | | | **Obese (BMI ≥ 30) n = 2817** | | | **p for contrast** |
| --- | --- | --- | --- | --- | --- | --- | --- |
|  | *β* | *95% Conf. Interval* | | *β* | *95% Conf. Interval* | |  |
| **Physical status, points, (SD)** | -0.68 | -1.72 | 0.36 | -2.16 | -4.96 | 0.65 | 0.332 |
| **Physical role, points, (SD)** | -1.91 | -4.20 | 0.38 | -8.76 | -14.89 | -2.62 | **0.04** |
| **Body pain, points, (SD)** | -0.81 | -2.43 | 0.81 | -5.58 | -9.94 | -1.23 | **0.044** |
| **General health, points, (SD)** | -1.12 | -2.19 | -0.06 | 0.31 | -2.55 | 3.18 | 0.356 |
| **Vitality, points, (SD)** | -0.30 | -1.48 | 0.89 | -0.63 | -3.82 | 2.56 | 0.848 |
| **Social role, points, (SD)** | 0.33 | -0.99 | 1.65 | -0.89 | -4.42 | 2.65 | 0.526 |
| **Emotional role, points, (SD)** | 0.22 | -1.77 | 2.21 | -0.46 | -5.77 | 4.85 | 0.815 |
| **Mental health, points, (SD)** | 0.23 | -0.86 | 1.32 | -2.83 | -5.76 | 0.10 | 0.055 |
| **Physical SF-36 summary, points, (SD)** | -0.57 | -1.09 | -0.06 | -1.53 | -2.90 | -0.15 | 0.205 |
| **Mental SF-36 summary, points, (SD)** | 0.34 | -0.30 | 0.97 | -0.18 | -1.88 | 1.52 | 0.575 |

**Suppl. Table 2c: stratified by high glucose levels prevalence:**

|  | **Low glucose (<110mg/dl) n = 1346** | | | **High glucose (≥110mg/dl) n = 2556** | | | **p for contrast** |
| --- | --- | --- | --- | --- | --- | --- | --- |
|  | *β* | *95% Conf. Interval* | | *β* | *95% Conf. Interval* | |  |
| **Physical status, points, (SD)** | -0.66 | -1.84 | 0.52 | -1.02 | -2.40 | 0.36 | 0.697 |
| **Physical role, points, (SD)** | -1.86 | -4.44 | 0.73 | -3.25 | -6.29 | -0.22 | 0.493 |
| **Body pain, points, (SD)** | -0.92 | -2.75 | 0.92 | -1.47 | -3.62 | 0.69 | 0.704 |
| **General health, points, (SD)** | -0.56 | -1.76 | 0.64 | -0.74 | -2.14 | 0.68 | 0.851 |
| **Vitality, points, (SD)** | 0.25 | -1.09 | 1.59 | -1.08 | -2.65 | 0.49 | 0.208 |
| **Social role, points, (SD)** | -0.05 | -1.54 | 1.44 | 0.71 | -1.04 | 2.46 | 0.514 |
| **Emotional role, points, (SD)** | -0.87 | -3.11 | 1.37 | 1.15 | -1.49 | 3.78 | 0.253 |
| **Mental health, points, (SD)** | -0.01 | -1.24 | 1.22 | -0.39 | -1.83 | 1.05 | 0.695 |
| **Physical SF-36 summary, points, (SD)** | -0.37 | -0.95 | 0.21 | -0.82 | -1.50 | -0.14 | 0.322 |
| **Mental SF-36 summary, points, (SD)** | 0.08 | -0.64 | 0.79 | 0.43 | -0.41 | 1.27 | 0.532 |

**Suppl. Table 2d: stratified by ALT/AST ratio group:**

|  | **Low ALT/AST ratio (<1.1) n = 1966** | | | **High ALT/AST ratio (≥1.1) n = 1936** | | | **p for contrast** |
| --- | --- | --- | --- | --- | --- | --- | --- |
|  | *β* | *95% Conf. Interval* | | *β* | *95% Conf. Interval* | |  |
| **Physical status, points, (SD)** | -0.95 | -1.90 | 0.01 | 1.64 | -4.57 | 7.84 | 0.42 |
| **Physical role, points, (SD)** | -1.90 | -3.99 | 0.19 | 1.11 | -12.42 | 14.64 | 0.666 |
| **Body pain, points, (SD)** | -1.21 | -2.69 | 0.27 | -8.08 | -17.71 | 1.55 | 0.167 |
| **General health, points, (SD)** | -0.53 | -1.50 | 0.44 | 2.61 | -3.75 | 8.96 | 0.339 |
| **Vitality, points, (SD)** | -0.25 | -1.32 | 0.83 | -0.84 | -7.90 | 6.22 | 0.871 |
| **Social role, points, (SD)** | 0.31 | -0.89 | 1.51 | -0.31 | -8.11 | 7.49 | 0.878 |
| **Emotional role, points, (SD)** | 0.01 | -1.80 | 1.82 | 0.83 | -10.85 | 12.51 | 0.892 |
| **Mental health, points, (SD)** | 0.04 | -0.95 | 1.03 | -0.42 | -6.91 | 6.07 | 0.89 |
| **Physical SF-36 summary, points, (SD)** | -0.56 | -1.02 | -0.09 | -0.26 | -3.31 | 2.80 | 0.849 |
| **Mental SF-36 summary, points, (SD)** | 0.29 | -0.29 | 0.87 | 0.01 | -3.75 | 3.77 | 0.885 |

**Supplementary table 3: Contrast of differences between Indeterminate probability of NAFLD (HSI < 36) and High probability of NAFLD (HSI > 36) of mixed multivariate model on the effect of Mediterranean diet adherence on SF-36 components after 1-year follow up**

**Suppl. Table 3a: stratified by age group:**

|  | **Age ≤65years n = 2002** | | | **Age >65years n = 1900** | | | **p for contrast** |
| --- | --- | --- | --- | --- | --- | --- | --- |
|  | *β* | *95% Conf. Interval* | | *β* | *95% Conf. Interval* | |  |
| **Physical status, points, (SD)** | 0.0010 | -0.0846 | 0.0865 | -0.0300 | -0.1123 | 0.0524 | 0.610 |
| **Physical role, points, (SD)** | -0.0440 | -0.2334 | 0.1454 | -0.2326 | -0.4150 | -0.0502 | 0.160 |
| **Body pain, points, (SD)** | -0.0595 | -0.1931 | 0.0742 | -0.1472 | -0.2759 | -0.0185 | 0.354 |
| **General health, points, (SD)** | -0.0154 | -0.1027 | 0.0720 | 0.0176 | -0.0665 | 0.1017 | 0.594 |
| **Vitality, points, (SD)** | -0.0521 | -0.1493 | 0.0452 | -0.0582 | -0.1518 | 0.0354 | 0.929 |
| **Social role, points, (SD)** | -0.0332 | -0.1422 | 0.0757 | 0.0334 | -0.0716 | 0.1383 | 0.388 |
| **Emotional role, points, (SD)** | 0.0667 | -0.0981 | 0.2316 | 0.0093 | -0.1495 | 0.1681 | 0.623 |
| **Mental health, points, (SD)** | -0.0136 | -0.1030 | 0.0758 | 0.0614 | -0.0246 | 0.1475 | 0.236 |
| **Physical SF-36 summary, points, (SD)** | -0.0163 | -0.0584 | 0.0259 | -0.0520 | -0.0925 | -0.0114 | 0.232 |
| **Mental SF-36 summary, points, (SD)** | 0.0026 | -0.0496 | 0.0548 | 0.0359 | -0.0143 | 0.0862 | 0.368 |

**Suppl. Table 3b: stratified by obesity prevalence:**

|  | **Overweight (BMI < 30) n = 1085** | | | **Obese (BMI ≥ 30) n = 2817** | | | **p for contrast** |
| --- | --- | --- | --- | --- | --- | --- | --- |
|  | *β* | *95% Conf. Interval* | | *β* | *95% Conf. Interval* | |  |
| **Physical status, points, (SD)** | 0.01 | -0.07 | 0.08 | -0.06 | -0.20 | 0.09 | 0.435 |
| **Physical role, points, (SD)** | -0.02 | -0.19 | 0.14 | -0.34 | -0.65 | -0.02 | 0.084 |
| **Body pain, points, (SD)** | -0.02 | -0.13 | 0.09 | -0.33 | -0.55 | -0.11 | **0.015** |
| **General health, points, (SD)** | 0.03 | -0.05 | 0.10 | -0.02 | -0.17 | 0.13 | 0.552 |
| **Vitality, points, (SD)** | -0.05 | -0.13 | 0.04 | -0.04 | -0.20 | 0.13 | 0.904 |
| **Social role, points, (SD)** | 0.04 | -0.05 | 0.13 | -0.06 | -0.25 | 0.12 | 0.326 |
| **Emotional role, points, (SD)** | 0.06 | -0.08 | 0.20 | -0.14 | -0.41 | 0.14 | 0.208 |
| **Mental health, points, (SD)** | 0.06 | -0.01 | 0.14 | -0.11 | -0.26 | 0.04 | **0.042** |
| **Physical SF-36 summary, points, (SD)** | -0.01 | -0.05 | 0.03 | -0.06 | -0.13 | 0.01 | 0.205 |
| **Mental SF-36 summary, points, (SD)** | 0.03 | -0.02 | 0.07 | -0.03 | -0.12 | 0.06 | 0.254 |

**Suppl. Table 3c: stratified by high glucose levels prevalence:**

|  | **Low glucose (<110mg/dl) n = 1346** | | | **High glucose (≥110mg/dl) n = 2556** | | | **p for contrast** |
| --- | --- | --- | --- | --- | --- | --- | --- |
|  | *β* | *95% Conf. Interval* | | *β* | *95% Conf. Interval* | |  |
| **Physical status, points, (SD)** | 0.02 | -0.06 | 0.10 | -0.07 | -0.16 | 0.02 | 0.133 |
| **Physical role, points, (SD)** | -0.07 | -0.25 | 0.10 | -0.26 | -0.47 | -0.06 | 0.168 |
| **Body pain, points, (SD)** | -0.08 | -0.20 | 0.05 | -0.13 | -0.27 | 0.02 | 0.608 |
| **General health, points, (SD)** | -0.01 | -0.09 | 0.07 | 0.01 | -0.08 | 0.11 | 0.744 |
| **Vitality, points, (SD)** | -0.06 | -0.15 | 0.03 | -0.06 | -0.17 | 0.04 | 0.939 |
| **Social role, points, (SD)** | 0.02 | -0.08 | 0.12 | -0.02 | -0.14 | 0.09 | 0.586 |
| **Emotional role, points, (SD)** | 0.05 | -0.11 | 0.20 | 0.00 | -0.17 | 0.18 | 0.721 |
| **Mental health, points, (SD)** | 0.07 | -0.02 | 0.15 | -0.03 | -0.13 | 0.07 | 0.135 |
| **Physical SF-36 summary, points, (SD)** | -0.02 | -0.06 | 0.02 | -0.05 | -0.10 | -0.01 | 0.381 |
| **Mental SF-36 summary, points, (SD)** | 0.03 | -0.02 | 0.07 | 0.01 | -0.05 | 0.06 | 0.625 |

**Suppl. Table 3d: stratified by ALT/AST ratio group:**

|  | **Low ALT/AST ratio (<1.1) n = 1966** | | | **High ALT/AST ratio (≥1.1) n = 1936** | | | **p for contrast** |
| --- | --- | --- | --- | --- | --- | --- | --- |
|  | *β* | *95% Conf. Interval* | | *β* | *95% Conf. Interval* | |  |
| **Physical status, points, (SD)** | -0.02 | -0.08 | 0.05 | -0.19 | -1.18 | 0.80 | 0.734 |
| **Physical role, points, (SD)** | -0.18 | -0.32 | -0.04 | -0.20 | -2.35 | 1.96 | 0.988 |
| **Body pain, points, (SD)** | -0.11 | -0.21 | -0.01 | 0.76 | -0.78 | 2.29 | 0.268 |
| **General health, points, (SD)** | 0.00 | -0.06 | 0.07 | -0.30 | -1.31 | 0.71 | 0.556 |
| **Vitality, points, (SD)** | -0.05 | -0.12 | 0.03 | -0.16 | -1.28 | 0.97 | 0.852 |
| **Social role, points, (SD)** | 0.01 | -0.07 | 0.09 | -0.12 | -1.36 | 1.12 | 0.836 |
| **Emotional role, points, (SD)** | 0.00 | -0.12 | 0.13 | 0.04 | -1.82 | 1.91 | 0.966 |
| **Mental health, points, (SD)** | 0.05 | -0.02 | 0.12 | -0.01 | -1.05 | 1.03 | 0.911 |
| **Physical SF-36 summary, points, (SD)** | -0.04 | -0.07 | -0.01 | -0.01 | -0.49 | 0.48 | 0.899 |
| **Mental SF-36 summary, points, (SD)** | 0.03 | -0.01 | 0.06 | -0.04 | -0.64 | 0.56 | 0.839 |

| **Suppl. Table 4. Linear trend of HSI quartiles of the Mediterranean diet adherence effect** | | | | | | | | | |
| --- | --- | --- | --- | --- | --- | --- | --- | --- | --- |
|  | **HSI Q1 n = 2040** | | **HSI Q2 n = 1946** | | **HSI Q3 n = 1914** | | **HSI Q4 n = 1904** | | **p for linear trend** |
|  | *β* | *SE* | *β* | *SE* | *β* | *SE* | *β* | *SE* |  |
| **Physical status, points, (SD)** | 0.0597 | 0.2136 | 0.1064 | 0.2168 | -0.0110 | 0.2193 | -0.3077 | 0.2290 | 0.216 |
| **Physical role, points, (SD)** | 0.6216 | 0.4770 | 1.2196 | 0.4856 | 0.0919 | 0.4901 | 0.0277 | 0.5101 | 0.186 |
| **Body pain, points, (SD)** | 0.2150 | 0.3356 | -0.2062 | 0.3412 | 0.3410 | 0.3447 | 0.0637 | 0.3593 | 0.952 |
| **General health, points, (SD)** | 0.0751 | 0.2181 | 0.6232 | 0.2213 | 0.7213 | 0.2240 | 0.5222 | 0.2339 | 0.153 |
| **Vitality, points, (SD)** | 0.2996 | 0.2430 | 0.4753 | 0.2467 | 0.0836 | 0.2496 | 0.0174 | 0.2606 | 0.270 |
| **Social role, points, (SD)** | 0.1839 | 0.2744 | 0.0499 | 0.2793 | 0.3080 | 0.2819 | 0.3356 | 0.2935 | 0.573 |
| **Emotional role, points, (SD)** | 0.2972 | 0.4159 | 1.2242 | 0.4241 | -0.4467 | 0.4274 | -0.0159 | 0.4443 | 0.173 |
| **Mental health, points, (SD)** | 0.2021 | 0.2233 | 0.3157 | 0.2267 | -0.1395 | 0.2294 | 0.3005 | 0.2395 | 0.877 |
| **Physical SF-36 summary, points, (SD)** | 0.0747 | 0.1057 | 0.0612 | 0.1073 | 0.2061 | 0.1086 | 0.0054 | 0.1133 | 0.897 |
| **Mental SF-36 summary, points, (SD)** | 0.1008 | 0.1309 | 0.2790 | 0.1331 | -0.1179 | 0.1345 | 0.1192 | 0.1401 | 0.571 |

| **Suppl. Table 5. Linear trend of HSI quartiles of the physical activity effect** | | | | | | | | | | |
| --- | --- | --- | --- | --- | --- | --- | --- | --- | --- | --- |
|  | **HSI Q1 n = 2040** | | **HSI Q2 n = 1946** | | **HSI Q3 n = 1914** | | **HSI Q4 n = 1904** | | **p for linear trend** | |
|  | *β* | *SE* | *β* | *SE* | *β* | *SE* | *β* | *SE* |  |  |
| **Physical status, points, (SD)** | 0.0182 | 0.0142 | 0.0123 | 0.0151 | 0.0270 | 0.0157 | 0.0116 | 0.0166 | 0.941 |  |
| **Physical role, points, (SD)** | 0.0995 | 0.0323 | 0.0491 | 0.0343 | 0.0474 | 0.0358 | -0.0234 | 0.0377 | **0.018** |  |
| **Body pain, points, (SD)** | 0.0664 | 0.0225 | 0.0168 | 0.0240 | 0.0244 | 0.0250 | -0.0050 | 0.0263 | 0.059 |  |
| **General health, points, (SD)** | 0.0066 | 0.0145 | 0.0063 | 0.0155 | 0.0383 | 0.0161 | 0.0021 | 0.0170 | 0.795 |  |
| **Vitality, points, (SD)** | 0.0157 | 0.0162 | 0.0130 | 0.0173 | -0.0122 | 0.0179 | -0.0162 | 0.0189 | 0.124 |  |
| **Social role, points, (SD)** | 0.0268 | 0.0185 | -0.0016 | 0.0197 | -0.0042 | 0.0206 | 0.0141 | 0.0217 | 0.653 |  |
| **Emotional role, points, (SD)** | -0.0198 | 0.0284 | 0.0102 | 0.0302 | 0.0150 | 0.0315 | 0.0059 | 0.0332 | 0.551 |  |
| **Mental health, points, (SD)** | 0.0047 | 0.0149 | 0.0057 | 0.0159 | -0.0220 | 0.0165 | 0.0041 | 0.0174 | 0.682 |  |
| **Physical SF-36 summary, points, (SD)** | 0.0237 | 0.0070 | 0.0081 | 0.0075 | 0.0183 | 0.0078 | -0.0017 | 0.0082 | 0.054 |  |
| **Mental SF-36 summary, points, (SD)** | -0.0070 | 0.0088 | 0.0002 | 0.0094 | -0.0125 | 0.0097 | 0.0021 | 0.0103 | 0.734 |  |
