# Supplementary material for: Role of NAFLD on the Health Related QoL Response to Lifestyle in Patients With Metabolic Syndrome: The PREDIMED Plus Cohort
Source: Front Endocrinol (Lausanne). 2022 Jun 29;13:868795. doi: 10.3389/fendo.2022.868795 (PMC9276971; doi:10.3389/fendo.2022.868795)

Supplementary Figure 1. Prevalence distribution of Metabolic syndrome features among HSI quartiles at baseline

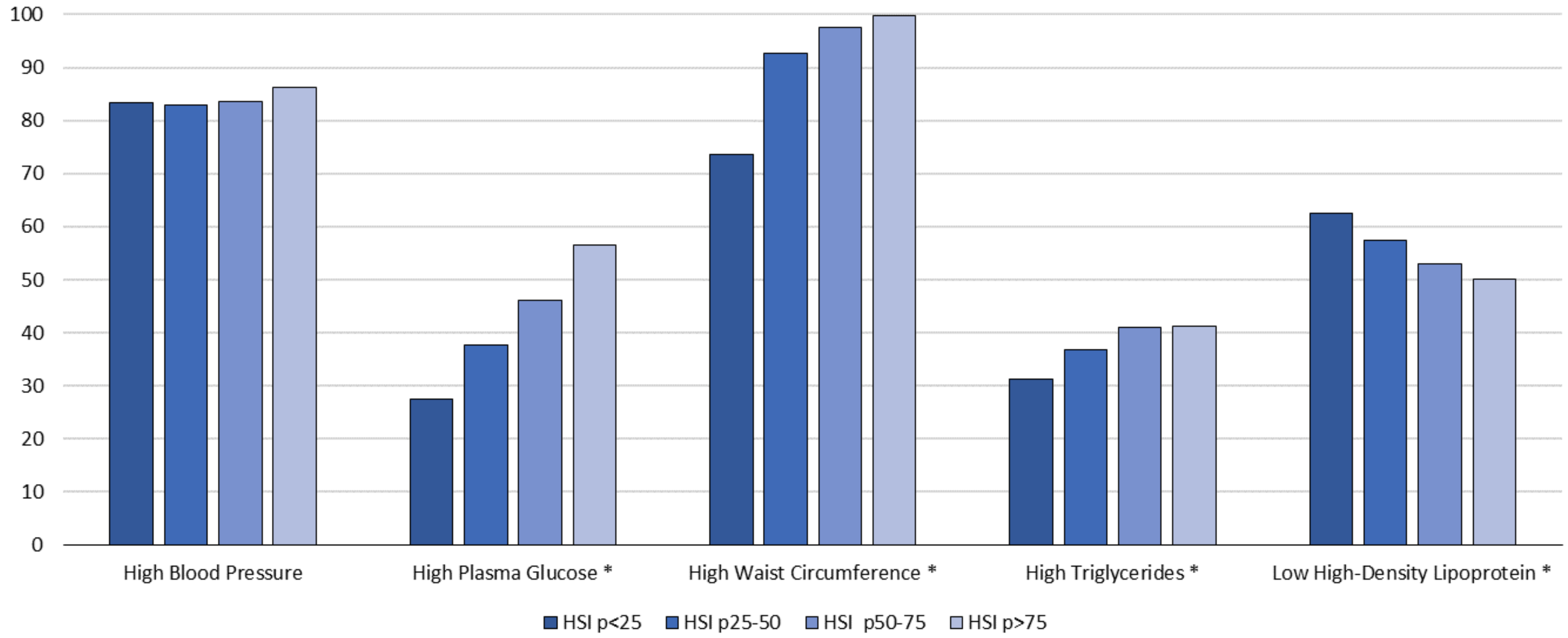

*HSI: Hepatic steatosis index; *p for trend < 0.001*

Supplementary figure 2. Clinical representation of predicted effect of lifestyle modification in terms of SF-36 PCS according to liver status.

Figure 2a. er-MeDiet adherence

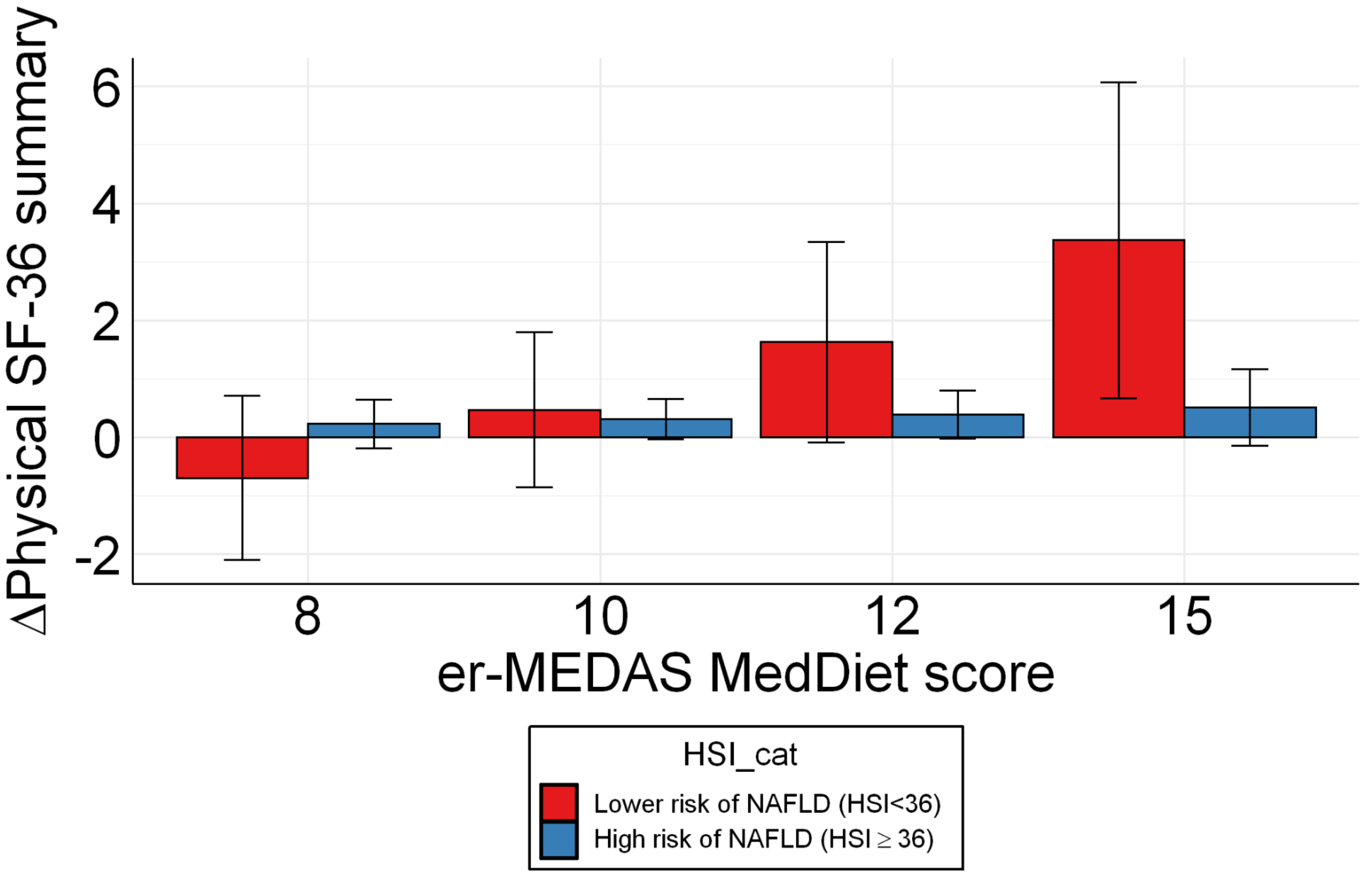

Figure 2b. Physical activity performance

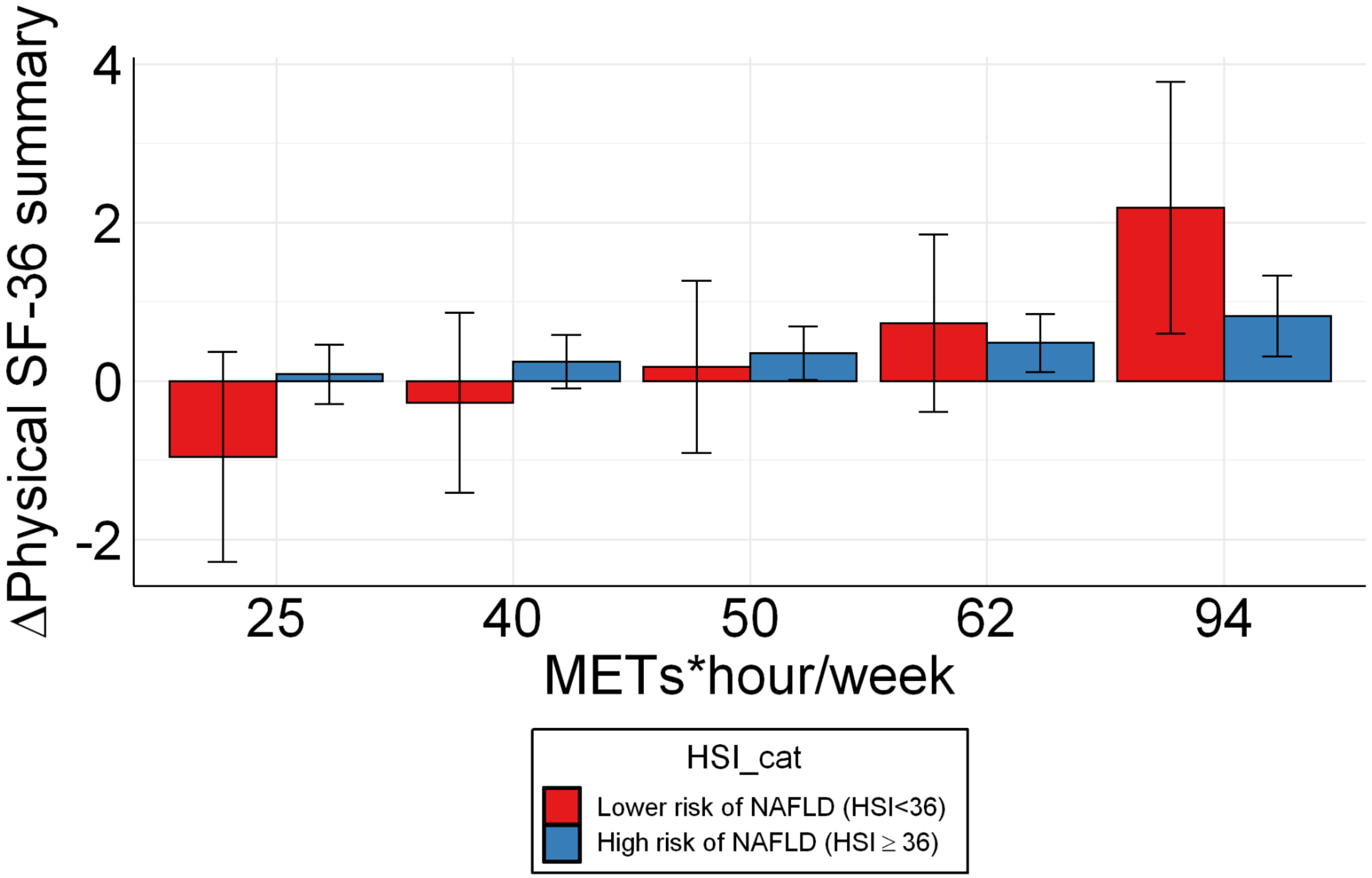

Supplement: Supplementary file 2 [file DataSheet_2.pdf]
